# Highly Efficient Solar-Light-Active Ag-Decorated g-C_3_N_4_ Composite Photocatalysts for the Degradation of Methyl Orange Dye

**DOI:** 10.3390/mi14071454

**Published:** 2023-07-20

**Authors:** Sakthivel Kumaravel, Chandramoorthy Chandrasatheesh, Govindasamy Palanisamy, Jintae Lee, Imran Hasan, Saranraj Kumaravel, Balakrishna Avula, Uma Devi Pongiya, Krishnakumar Balu

**Affiliations:** 1 Department of Environmental Engineering, Korea Maritime and Ocean University, Busan 49112, Republic of Korea; 2 Department of Applied Science and Technology, AC Tech, Anna University, Chennai 600025, Tamil Nadu, India; 3 School of Chemical Engineering, Yeungnam University, 280 Daehak-Ro, Gyeongsan 38541, Republic of Korea; 4 Department of Chemistry, College of Science, King Saud University, Riyadh 11451, Saudi Arabia; 5 Department of Electrical Engineering, National Taipei University of Technology, Taipei 10608, Taiwan; 6 Department of Chemistry, Rajeev Gandhi Memorial College of Engineering and Technology (Autonomous), Nandyal 518501, Andhra Pradesh, India; 7 Department of Biochemistry, Dhanalakshmi Srinivasan College of Arts and Science for Women (Autonomous), Perambalur 621212, Tamil Nadu, India; 8 Departamento de Ingeniería y Ciencia de los Materiales y del Transporte, E.T.S. de Ingenieros, Universidad de Sevilla, Avda. Camino de los Descubrimientos s/n., 41092 Sevilla, Spain; 9 Department of Chemistry, Saveetha School of Engineering, Saveetha Institute of Medical and Technical Sciences, Saveetha University, Chennai 602105, Tamil Nadu, India

**Keywords:** methyl orange, gC_3_N_4_, Ag/gC_3_N_4_, photocatalyst, photodegradation

## Abstract

In this study, we utilized calcination and simple impregnation methods to successfully fabricate bare g-C_3_N_4_ (GCN) and x% Ag/g-C_3_N_4_ (x% AgGCN) composite photocatalysts with various weight percentages (x = 1, 3, 5, and 7 wt.%). The synthesized bare and composite photocatalysts were analyzed to illustrate their phase formation, functional group, morphology, and optical properties utilizing XRD, FT-IR, UV-Vis DRS, PL, FE-SEM, and the EDS. The photodegradation rate of MO under solar light irradiation was measured, and the 5% AgGCN composite photocatalyst showed higher photocatalytic activity (99%), which is very high compared to other bare and composite photocatalysts. The MO dye degradation rate constant with the 5% AgGCN photocatalyst exhibits 14.83 times better photocatalytic activity compared to the bare GCN catalyst. This photocatalyst showed good efficiency in the degradation of MO dye and demonstrated cycling stability even in the 5th successive photocatalytic reaction cycle. The higher photocatalytic activity of the 5% AgGCN composite catalyst for the degradation of MO dye is due to the interaction of Ag with GCN and the localized surface plasmon resonance (SPR) effect of Ag. The scavenger study results indicate that O_2_^●−^ radicals play a major role in MO dye degradation. A possible charge-transfer mechanism is proposed to explain the solar-light-driven photocatalyst of GCN.

## 1. Introduction

As industrial and agricultural areas were developed, environmental pollution became more serious. Organic pollutants are often discharged into wastewater in the form of dyes. Organic pigments and dyes used in industries such as leather, textiles, paper, cosmetics, and printing can cause water pollution [1,2]. Dye color is a major attraction for all textile materials and is therefore widely used in these industries [3]. The textile dyeing industry has been identified as a major source of water pollution, with typically 15–50% of textile dyes being discarded, contaminating natural water bodies [4]. When wastewater containing dyes is introduced into water sources, it can be hazardous as it can introduce harmful chemicals and contaminants into the environment. Methyl orange (MO) is a water-soluble organic synthetic dye with an azo anion structure, which exhibits a bright orange color when dissolved in water and is widely used in dye synthesis and industrial application processes [5]. However, due to its high coloring properties and the presence of aromatic groups and a –N=N– group, which are considered highly toxic, carcinogenic, and teratogenic, waste from printing and dyeing factories is a great threat to the environment and living things [6]. There is an urgent need to deal with organic pollution in water. There are several methods to treat wastewater, including membrane filtration, advanced oxidation, electrochemical oxidation, photoelectrochemical oxidation, and ozonation [7,8,9,10,11,12]. However, most of these conventional methods are not considered environmentally friendly and suffer from many drawbacks such as high cost, low efficiency, toxic sludge, etc. [13]. The most economical and reliable treatment technology for the degradation of harmful dyes present in industrial wastewater is the advanced oxidation process (AOP) [14]. Among different AOPs, sunlight-induced photocatalytic decomposition has emerged as an environmentally friendly, energy-saving, and highly effective process to remove organic pollutants from water [15,16].

Recently, the photodegradation of organic contaminants in aqueous solutions has been found to be an effective process in utilizing semiconductor materials. Major semiconductor photocatalysts include graphite nitride (GCN), metal oxides, metal sulfides, etc. [17]. GCN has been extensively investigated as a very interesting photocatalyst due to its unique properties such as being metal-free, non-toxic, and easy to prepare, as well as having a suitable bandgap, having good chemical/physical properties, and being inexpensive [18]. Since it has low sunlight utilization efficiency and fast recombination of photo-generated e^−^/h^+^ pairs, its photocatalytic activity is still low, making it difficult to use in real life [19,20,21]. In addition, various techniques such as metallic/non-metallic doping, noble metal deposition, and combination with semiconductors have been investigated to address the problem of the low optical absorption and fast recombination of photo–generated electron–hole pairs in GCN [22]. Due to the surface plasmon resonance (SPR) phenomenon, noble metal deposition has received the most attention among these techniques [23]. Silver nanoparticles (Ag NPs) have attracted more interest because they are cheaper than other precious metals such as gold (Au), platinum (Pt), and ruthenium (Ru) [24]. The addition of Ag has been shown to greatly enhance the photolytic capacity of the materials through surface charging, enhanced light absorption capacity, and synergistic effects [25]. The aim of this research is to develop metal–oxide-based composites that are environmentally friendly and can efficiently remove organic contaminants. In this study, bare GCN and x% AgGCN composite photocatalysts with various weight percentages (x = 1, 3, 5, and 7 wt.%) were successfully prepared by the calcination and simple impregnation methods. All the fabricated materials were characterized by several techniques. We evaluated the degradation performance of MO dyes over bare GCN and x% AgGCN photocatalysts under solar light illumination. The 5% AgGCN composite photocatalyst maintains good stability and photocatalytic activity even after degradation for five cycles. Finally, a possible photocatalytic reaction mechanism is proposed.

## 2. Experimental Section

### 2.1. Chemicals and Reagents

Analytical grade melamine (C_3_H_6_N_6_, 99%), ethanol (≥99.9%), silver nitrate (AgNO_3_, ≥99%), and methyl orange (MO) were purchased from Deoksan Pharmaceutical Co., Ltd., Deoksan, Republic of Korea. Millipore water was used for washing and papering the dye solution.

### 2.2. Synthesis of GCN and x% AgGCN Composite Catalysts

The bare GCN was fabricated by the calcination method [26]. First, 2 g of melamine was heated in a covered crucible at 500 °C for 3 h with a heating rate of 3 °C min^−1^. The reaction was cooled to atmospheric temperature after which the yellow solid compound was ground into a fine powder. The fabricated g-C_3_N_4_ catalyst is called GCN. The impregnation method was used to fabricate AgGCN composite photocatalysts. First, 1 g of GCN nanosheets was dispersed in a 30 mL solution with a 1:1 ratio of water and ethanol for 30 min with constant stirring. After the solution was stirred for 1 h the calculated amount of 5% AgNO_3_ was added to the above solution. Finally, the resulting mixture was dried at 70 °C for 6 h followed by calcination at 350 °C for 2 h. Different percentages of x% Ag (x = 1, 3, 5, and 7% wt.%) were added to g-C_3_N_4_, resulting in samples labeled as 1% AgGCN, 3% AgGCN, 5% AgGCN, and 7% AgGCN, respectively.

### 2.3. Characterizations of Materials

The phase formation and crystal structure of the fabricated bare and composite photocatalysts were analyzed by XRD (Miniflex, Rigaku, Tokyo, Japan). FT-IR spectroscopy was performed using a Thermo Fisher iS50 spectrometer (Thermo Fisher Scientific India Pvt Ltd., Mumbai, India). UV-DRS analysis was carried out using (JASCO-V750, Anatek Services Private Ltd., Mumbai, India). The morphology of the bare GCN and 5% AgGCN composite catalyst was characterized using scanning electron microscopy (SEM, Hitachi S-4800, Tokyo, Japan) coupled with an energy dispersive spectrum (EDS) analyzer.

### 2.4. Photocatalytic Activity Measurement

All the samples were evaluated as synthesized for photocatalytic activity by measuring their ability to degrade methyl orange in an aqueous solution under sunlight irradiation. Photocatalytic experiments were performed using 30 mg of bare GCN and x% AgGCN (x = 1, 3, 5, and 7 wt.%) composite photocatalysts in 100 mL of 20 ppm MO aqueous dye solution. The dye solution was kept stirred in the dark for 30 min before irradiation. The dye solution was exposed to sunlight and constantly stirred. An amount 3 mL of the MO dye solution was taken at each subsequent 15 min interval to determine the concentration of degraded organic contaminants. A centrifugation process can be used to separate the photocatalyst from the dye solution. The UV-visible spectrophotometer was found to be an effective tool for estimating concentrations of dye solutions by recording the intensity of the absorption band of MO at 464 nm. The intensity of solar light measured with a TES Datalogging solar power meter was found to be 95 mW/cm^2^. The % degradation, apparent rate constant (k_app_), and half-life period (t_1/2_) were determined using the following equations (Equations (1)–(3)) [27]:

(1) $$\mathrm{Degradation}\;(\%)=[1-\frac{C_{t}}{C_{0}}]\times 100$$

(2) $$\mathrm{The}\;\mathrm{apparent}\;\mathrm{rate}\;\mathrm{constant}\;(k_{\mathrm{app}})=\frac{1}{t}\;\ln\;\frac{C_{0}}{C_{t}}$$

(3) $$\mathrm{Half}-\mathrm{life}\;\mathrm{period}\;(t_{1/2})=\frac{0.693}{k_{app}}$$

C_0_ represents the initial concentration, while C_t_ represents the dye’s concentration at time t.

## 3. Result and Discussion

### 3.1. XRD Analysis

**Figure 1:**
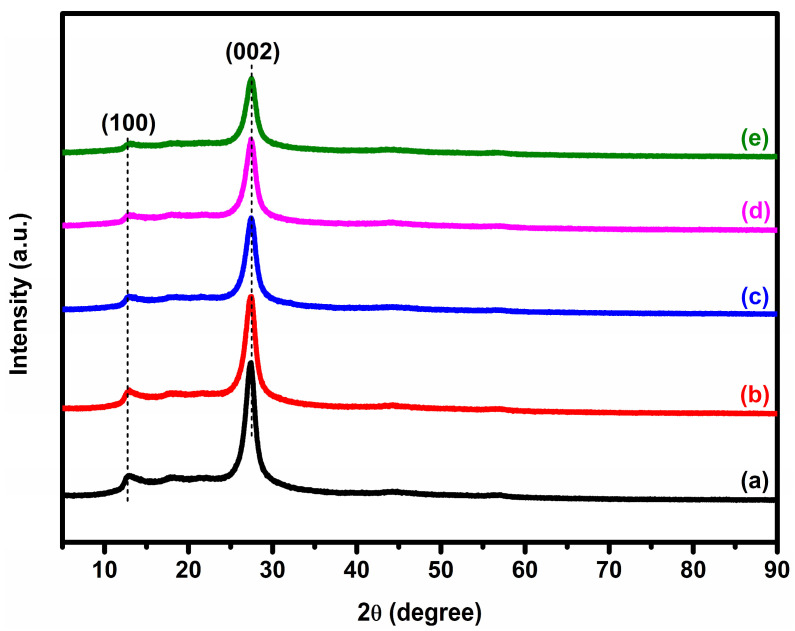
XRD patterns of (a) GCN, (b) 1% AgGCN, (c) 3% AgGCN, (d) 5% AgGCN, and (e) 7% AgGCN composite photocatalysts.

XRD measurements were used to study the purity and phase formation of the as-prepared materials. Figure 1 shows the XRD patterns of bare GCN and x% Ag-impregnated GCN (x = 1, 3, 5, and 7 wt.%) composite photocatalysts. The XRD patterns of both the pure GCN and x% AgGCN composites displayed two major peaks at 12.9° and 27.47°, characteristic of GCN (JCPDS 87-1526). These findings are in good agreement with previous XRD patterns [28,29].

The diffraction peaks at 12.9° and 27.47° correspond to the (100) and (002) planes of s-triazine, respectively, with a planar distance of 0.67 nm and 0.32 nm reflective of its in-plane structural repeating unit and structural packing arrangement [30]. The presence of Ag species did not affect the crystal structure of the GCN photocatalyst, as no prominent diffraction peaks of other phases or contaminants were found in the composite sample [31]. The diffraction intensity of the 27.47° peak decreases with increasing Ag content on GCN. This results in good agreement with previous literature reports [29,31]. Note that the diffraction peaks related to Ag in the AgGCN composite photocatalyst are not present in all XRD patterns. This could be attributed to the low Ag loading due to the high dilution effect of Ag on the GCN surface, which resulted in XRD analysis results below the detection limit [29,31]. Accordingly, the SEM/EDS analysis shows that there is Ag loading on the GCN surface.

### 3.2. FT-IR Analysis

**Figure 2:**
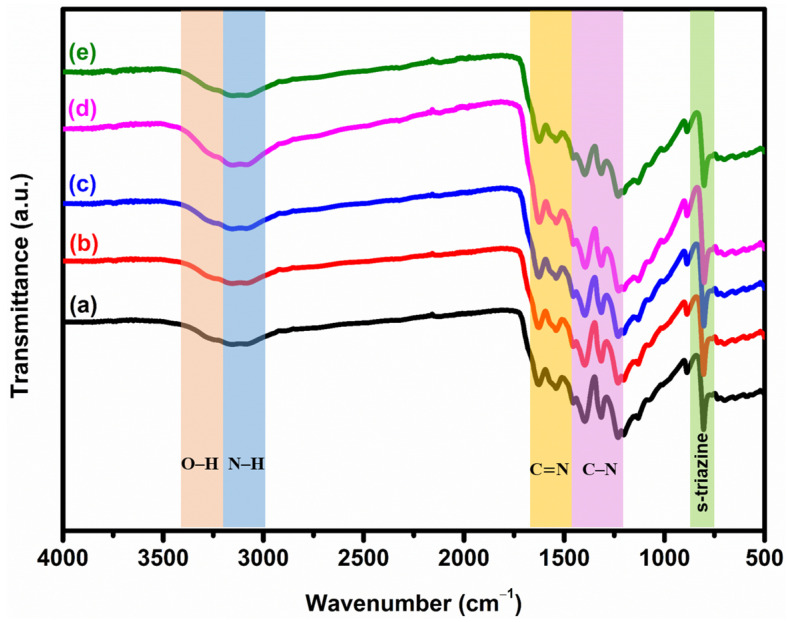
FT-IR spectra of (a) GCN, (b) 1% AgGCN, (c) 3% AgGCN, (d) 5% AgGCN, and (e) 7% AgGCN composite photocatalysts.

Figure 2 shows the FT–IR spectra of the surface characterization of as-prepared bare GCN and x% AgGCN (x = 1, 3, 5, and 7 wt.%) composite photocatalysts. Aromatic C=N and C–N stretching vibrations involve absorption bands at 1242, 1327, 1568, 1408, and 1639 cm^−1^ [32]. The bending vibration of the s–triazine unit is represented by a peak at 809 cm^−1^ [33]. The peaks between 3000 and 3300 cm^−1^ are attributed to stretching vibrations of N–H and O–H groups [34,35,36,37,38,39]. After the addition of Ag, all these characteristic FT–IR peaks indicate that the general structure of GCN is unchanged [40]. The peak intensity of the FT–IR spectra slightly decreased with increasing Ag loading compared to the spectra of bare GCN. This result suggests that the Ag is highly dispersed on the GCN surface. This finding is consistent with previous studies [29,41].

### 3.3. UV-DRS Analysis

**Figure 3:**
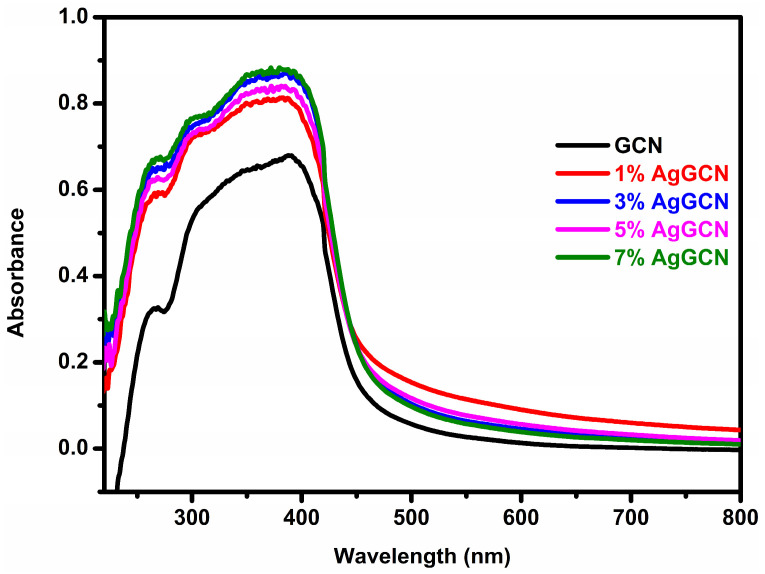
UV-DRS of bare and composite photocatalysts.

The optical properties of all the fabricated materials were investigated from UV-Vis diffuse reflectance spectra. The UV–Vis DRS spectra of the bare GCN and x% AgGCN (x = 1, 3, 5, and 7 wt.%) composite photocatalysts are shown in Figure 3. A typical semiconductor absorption feature can be seen in the bare GCN. The spectrum reveals that the optical absorption ranges from ultraviolet to visible light, with absorption bands ranging from 400 to 800 nm, corresponding to the characteristic bandgap of pure GCN. The visible light absorption intensity of the AgGCN photocatalyst is further enhanced by the surface plasmon resonance (SPR) of Ag and is higher than that of pristine GCN [42]. AgGCN photocatalysts with strong visible light responsiveness enhance photocatalytic activity in direct solar light. The bandgaps of bare GCN and x% AgGCN composite catalysts were determined using the formula Eg = 1240/λ, where λ is the cutoff wavelength [43]. The bandgap values of materials were calculated to be 2.60, 2.35, 2.34, 2.30, and 2.36 eV for the pure GCN, 1% AgGCN, 3% AgGCN, 5% AgGCN, and 7% AgGCN composite photocatalysts, respectively.

### 3.4. SEM/EDS Analysis

**Figure 4:**
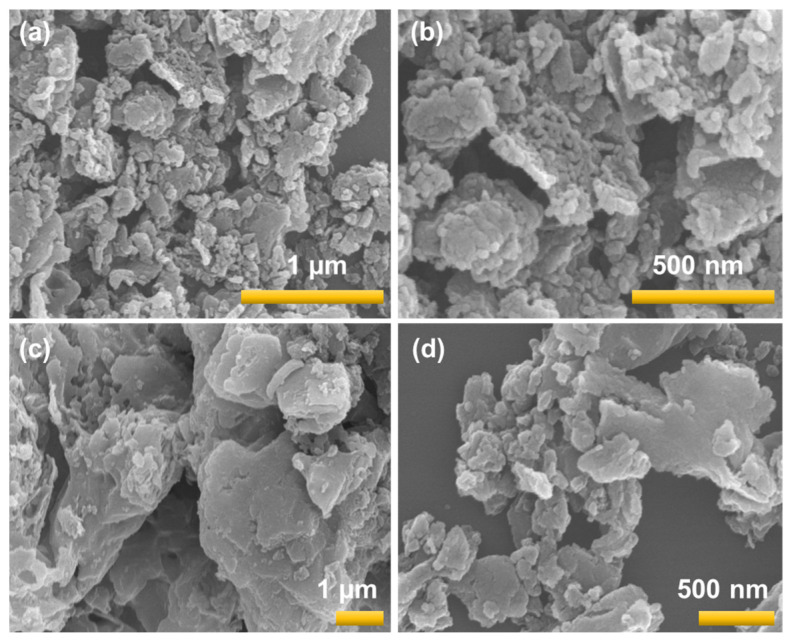
FE-SEM image of (**a**,**b**) GCN and (**c**,**d**) 5%AgGCN composite photocatalysts (yellow line is scale bar).

Figure 4a–d shows the FE-SEM image of the bare GCN and 5% AgGCN composite photocatalysts. As shown in Figure 4a–b, the bare GCN revealed a layered structure of irregularly shaped sheets made from melamine. The induction of Ag leads to the formation of similar sheets like layered structures and some agglomeration structure was observed. This was similarly observed in previous literature reports [31].

**Figure 5:**
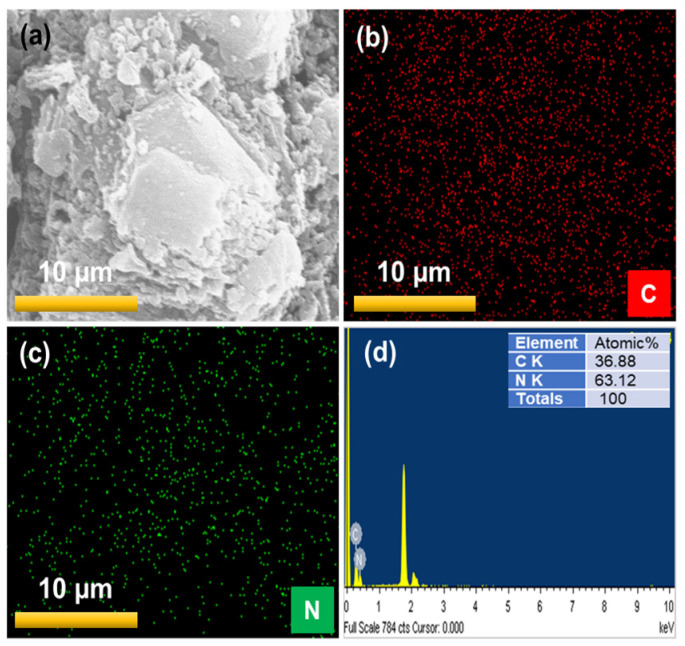
Elemental mapping analysis of (**a**) SEM image, (**b**) C, (**c**) N, and (**d**) energy dispersive spectrum of bare GCN photocatalyst.

**Figure 6:**
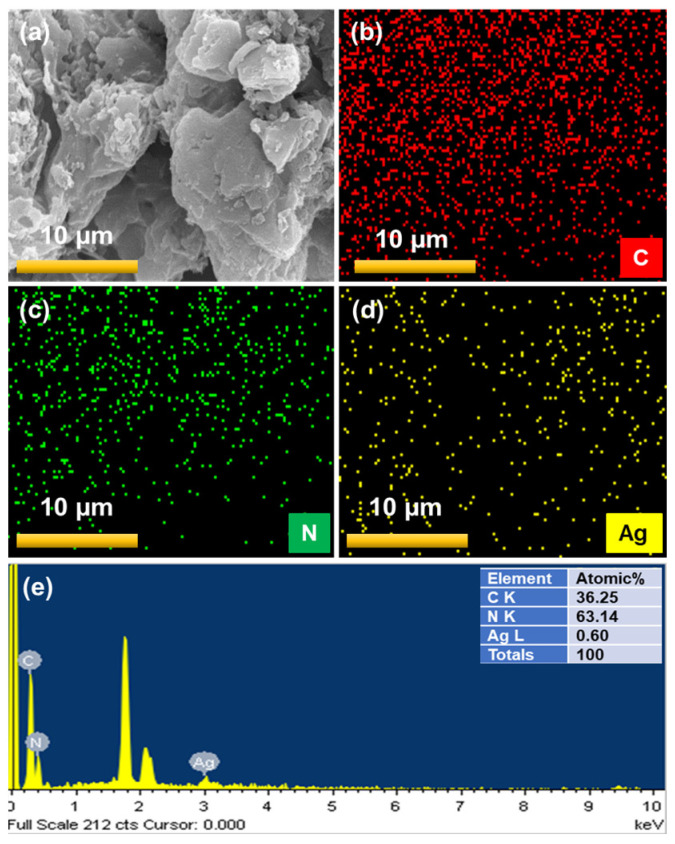
Elemental mapping analysis of (**a**) SEM image, (**b**) C, (**c**) N, (**d**) Ag, and (**e**) energy dispersive spectrum of 5% AgGCN composite photocatalyst.

The chemical composition of bare GCN and the most active 5% AgGCN composite catalysts were recorded using the EDS. Figure 5a–d shows the SEM image, elemental mapping, and EDS spectrum of the bare GCN photocatalyst. The elemental mapping of the 5% AgGCN nanocomposite reveals that the C, N, and Ag species are shown in Figure 6a–d. The Ag is uniformly distributed throughout the GCN surface. The chemical compositions of 5% AgGCN composite photocatalysts containing C, N, and Ag have been successfully evaluated with the EDS and are presented in the table (inset) shown in Figure 6e. This result suggests that the sample is of high purity and free of other impurities, providing evidence that the 5% Ag NPs were successfully assembled with GCN nanosheets.

The physico-chemical characterization reveals the successful formation of AgGCN composite photocatalysts. From XRD, the diffraction intensity of the 27.47° peak decreases with increasing Ag content on GCN. However, in the FT-IR study, after the addition of Ag, all the characteristic FT-IR peaks of GCN are unchanged. From SEM analysis, the bare GCN revealed a layered structure with irregularly shaped sheets, whereas AgGCN leads to the formation of similar sheets like layered structures with some agglomeration. The EDS and elemental mapping confirm the presence of Ag in AgGCN. The visible light absorption intensity of AgGCN photocatalysts is higher than that of pristine GCN due to the surface plasmon resonance (SPR) of Ag which makes this photocatalyst more active under solar light.

### 3.5. Photocatalytic Activity of Bare GCN and x% AgGCN Composite Photocatalysts

**Figure 7:**
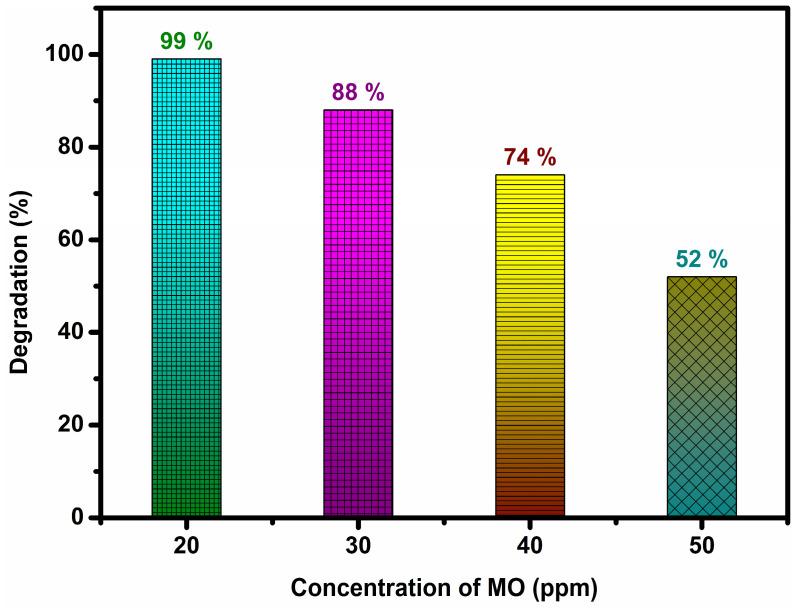
Effect of initial dye concentration on the degradation of MO dye over 5% AgGCN composite photocatalyst.

To investigate the effect of initial dye concentration on degradation rate, the experiments were performed using 5% AgGCN (30 mg) for 75 min. The dye concentration was varied from 20 to 50 ppm. The corresponding results are shown in Figure 7. As the dye concentration increased from 20 ppm to 50 ppm, the rate of MO degradation decreased significantly from 99% to 52%. The presence of so many dye molecules prevent light reaching across the catalyst surface [1]. The initial dye concentration was optimized at 20 ppm and was used for further studies.

**Figure 8:**
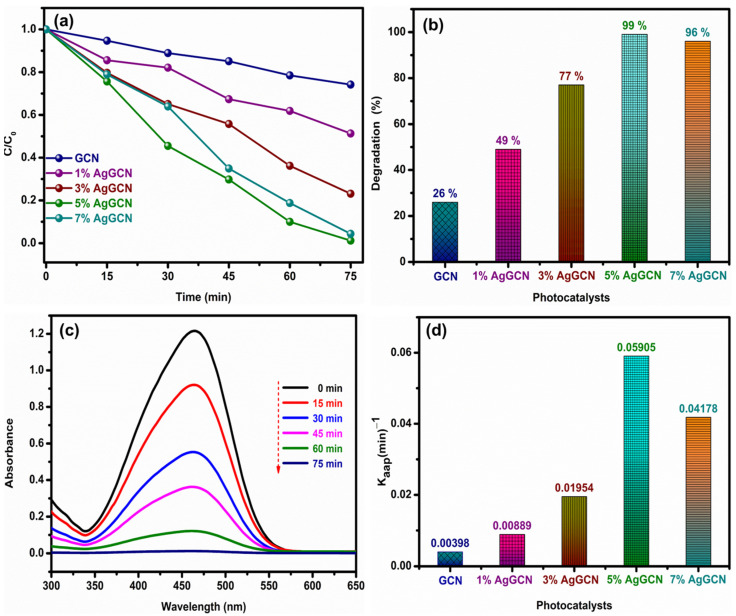
(**a**) Photocatalytic degradation of MO dye, (**b**) degradation efficiency at 75 min, (**c**) time dependence absorption spectra of MO in the presence of 5% AgGCN composite, and (**d**) rate constant values of bare and composite catalysts at 75 min (reaction conditions = 20 ppm of MO dye, 30 mg of catalyst weight, pH = ~4.5 (as such, dye pH), irradiation is solar light).

Figure 8a displays the photocatalytic performance of the prepared bare GCN and x% AgGCN composite photocatalysts in the degradation of the methyl orange dye aqueous solution under solar irradiation. As a result of solar light irradiation, the experiments demonstrated that the dye solution concentration did not change in the absence of the photocatalyst and revealed that MO dye is not degraded by solar irradiation without a photocatalyst. It is clear that direct photolysis is not powerful enough to effectively degrade aqueous MO dyes. Before the photocatalytic process, the suspension was placed in the dark, the adsorption–desorption equilibrium was measured, and then the sunlight irradiation was started. The bare GCN photocatalyst showed that 26% of the MO dye was degraded after 75 min of sunlight irradiation. Figure 8b reveals that the degradation activities of the prepared 1%, 3%, 5%, and 7% AgGCN composite photocatalysts were 49%, 77%, 99%, and 96%, respectively. Both the 5% AgGCN and 7%AgGCN composites photocatalysts showed significantly enhanced photocatalytic degradation of MO dyes under solar light irradiation when compared with other fabricated bare and composite photocatalysts. The efficiency of the fabricated photocatalysts for the degradation of MO dye is in the order of 5% AgGCN > 7% AgGCN > 3% AgGCN > 1% AgGCN > GCN. The photocatalytic efficacy is improved after Ag modification, which has a significant effect on carrier charge transfer. Figure 8c displays the gradual degradation of the MO dye solution in the 5% AgGCN composite photocatalyst with increasing irradiation time (15 min constant interval), as evidenced by the decrease in the maximum absorption band intensity at 464 nm.

Figure 8d shows the corresponding results of the K_aap_ value of MO dye degradation using the resulting bare GCN and x% AgGCN composite photocatalysts. As shown in Figure 8d, 5% AgGCN composite photocatalysts have the highest rate constant of 0.05905 min^−1^, which is 14.83, 6.63, 3.02, and 1.41 times higher than that of pure GCN (0.00398 min^−1^), 1% AgGCN (0.00889 min^−1^), 3% AgGCN (0.01954 min^−1^), and 7% AgGCN (0.04178 min^−1^), respectively. The calculated half-life period (t_1/2_) values in the MO dye degradation using the 5% AgGCN composite catalyst (11.73 min) were found to be higher than that of 7% AgGCN (16.58 min), 3% AgGCN (34.47 min), 1%AgGCN (77.88 min), and bare GCN (173.95). All the fabricated materials were well linearly fitted with the relationship coefficient value (R^2^ ≈ 1) and the data obtained were fitted to a *pseudo*-first-order kinetic model. The calculated R^2^ values for the as-obtained bare GCN and 1%, 3%, 5%, and 7% AgGCN composite photocatalysts throughout the MO dye degradation reaction were 0.9955, 0.9797, 0.9720, 0.8647, and 0.8838, respectively.

**Figure 9:**
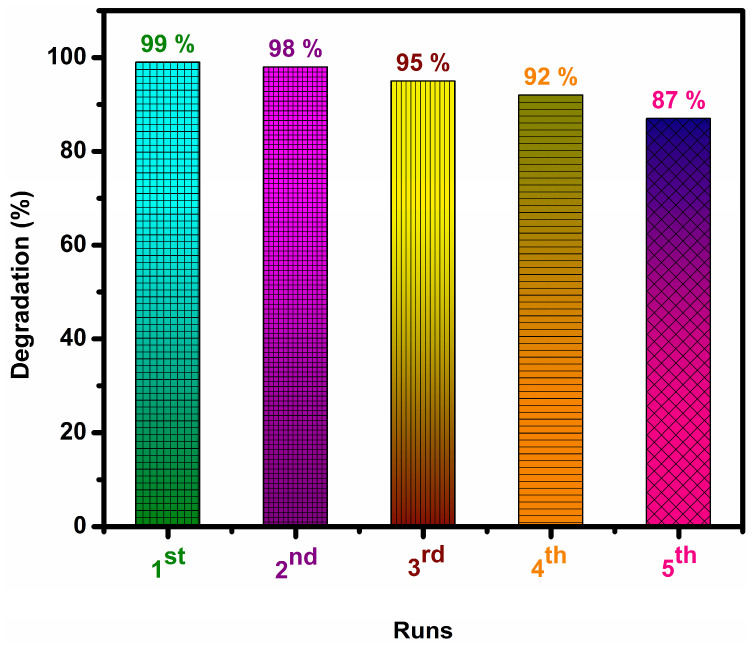
Recyclability test of 5% AgGCN composite photocatalyst.

An important factor to consider when developing a practical strategy is the photostability of the catalyst. Figure 9 shows the recycling stability test of the most active 5% AgGCN composite photocatalyst. The photocatalytic degradation activity of the 5% AgGCN catalyst showed sustained performance of the MO dye over five consecutive recycling cycles with the same solar light illumination.

The 5% AgGCN nanocomposite appears to have good stability and reusability, as only negligible efficiency loss is observed after the fourth and fifth photocatalysis cycles. A slight decrease in photolytic performance (99–87%) occurred. The present study is compared with recently reported studies on the photocatalytic degradation of AgGCN composite catalysts given in Table 1.

### 3.6. Catalytic Mechanism

**Figure 10:**
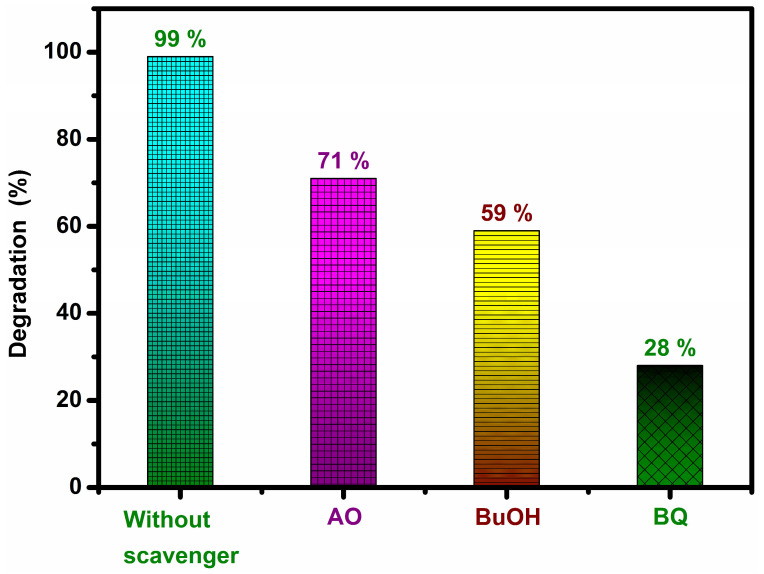
Effect of different scavenger experiments for the degradation of MO with 5% AgGCN composite photocatalyst.

The as-obtained most active 5% AgGCN composite photocatalyst has the potential to treat wastewater in an environmentally friendly manner. The involvement of key reactive species in photocatalytic systems was studied to determine the effects of different radical scavengers on the photodegradation of MO dyes (Figure 10). In general, the photodegradation mechanism involving multiple reactive species was performed using ammonium oxalate (AO), *tert*-butanol (BuOH), and *p*-benzoquinone (BQ) as trapping agents for h^+^, ^●^OH, and O_2_^●−^ [1]. Meanwhile, BQ was used for the O_2_^●−^ scavenger, and the decomposition performance was lower compared to other scavengers. As evident from Figure 10, the photocatalysis efficiency of 5% AgGCN was significantly reduced in the presence of BQ. This suggests that O_2_^●−^ was the predominant reactive species. The introduction of the ^●^OH and h^+^ scavengers BuOH and AO reduced the photocatalytic activity of the 5%AgGCN composite catalyst to 59% and 71%, respectively, from 99% (without scavenger). The scavenger study results indicate that O_2_^●−^ radicals are the main contributors to the enhanced photocatalytic performance of the system [42].

**Figure 11:**
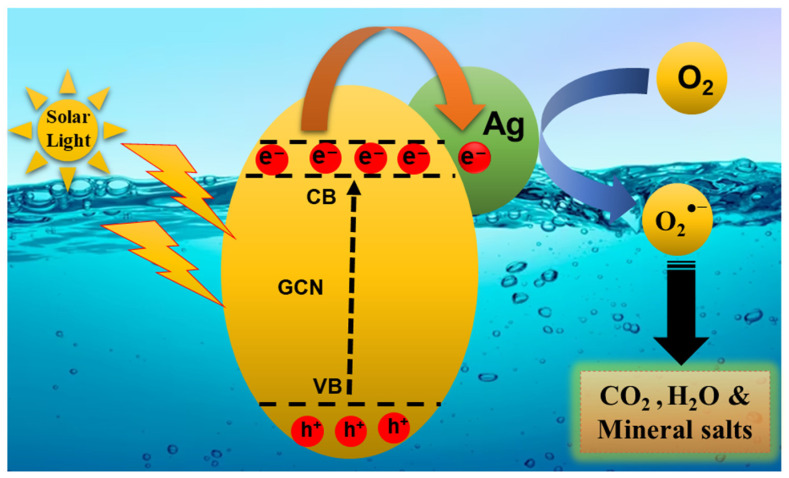
A possible photocatalytic mechanism using the 5% AgGCN composite photocatalyst.

Based on the above scavenger results, a possible photocatalytic mechanism of the MO dye under sunlight with the most active 5% AgGCN composite photocatalyst was proposed and is shown in Figure 11. The well-known semiconductor GCN material is stimulated to produce both holes and electrons when exposed to solar light. Modification of Ag enhances the photocatalytic performance of GCN through a combination of both the surface plasmon resonance (SPR) effect of Ag and the reduced recombination rate of photogenerated e^−^/h^+^ pairs [19].

The most active 5%AgGCN material produces isolated electron–hole pairs when exposed to sunlight, and electrons are excited into the conduction band (CB) of GCN while holes remain in the valence band (VB). The high Schottky barrier of Ag allows electron transfer from Ag and participation in the reduction reaction at the photocatalytic surface. We investigated the generation of electrons by plasmon-excited Ag and photoexcited GCN sheets. These electrons can react with O_2_ to form O_2_^●−^ radicals and catalyze the degradation of the MO dye molecule into CO_2_ and H_2_O.

## 4. Conclusions

In summary, bare GCN and x% AgGCN composite photocatalysts with various weight percentages (x = 1, 3, 5, and 7 wt.%) were successfully prepared by calcination and simple impregnation methods. The most active 5% AgGCN composite photocatalyst has been demonstrated to have significantly enhanced photocatalytic performance for MO dye degradation compared to other bare and composite photocatalysts. This enhances the photocatalytic performance of 5% AgGCN through a combination of both the surface plasmon resonance (SPR) effect of Ag and the reduced recombination rate of photogenerated e−/h+ pairs. The rate constant value of the 5% AgGCN composite photocatalyst is 14.83 times higher than that of the bare GCN catalyst. Moreover, the photocatalytic degradation performance of the fabricated composite material shows no significant signs of declining even after the fifth cycle. The degradation of dye-contaminated wastewater from the textile industry can be achieved using the high performance of the 5% AgGCN composite photocatalyst.

## Figures and Tables

**Table 1:** Photocatalytic degradation of MO dye by various composites of Ag and GCN.

S. No.	Composites	Pollutants and Concentration	Light Source and Irradiation Time	Efficiency (%)	Ref.
1	PPy@Ag/g-C_3_N_4_	MO, 10 ppm	Visible, 60 min	89%	[41]
2	1.0 wt%Ag/g-C_3_N_4_	MO, 10 ppm	Visible, 180 min	100%	[31]
3	Ag/g-C_3_N_4_-2	RhB, 10 ppm	Visible, 120 min	98%	[42]
4	3% Ag/g-C_3_N_4_	RhB, 10 ppm	Simulated sunlight, 120 min	100%	[29]
5	Ag-B-NS-gC_3_N_4_	RhB, 10 ppm	Visible, 60 min	99%	[44]
6	Ag/CN-8	MO, 10 ppm	Visible, 120 min	98.7%	[45]
**7**	**5% AgGCN**	**MO, 20 ppm**	**Solar, 75 min**	**99%**	**This work**

## Data Availability

The datasets used or analyzed during the current study are available from the corresponding author upon reasonable request.
